# The Groan of the Willing Horse

**Published:** 1886-10-16

**Authors:** 


### The Groan of the Willing Horse.

The Willing Horse.

It is a familiar saying in agricultural districts that "the willing horse may work." The downright worker thereby expresses his contempt for the shuffler who stands by with his hands in his pockets. It is the experience of every hospital that the horse which is willing is promptly left to do all the work, and that, unfortunately, willing horses are but few. It is quite clear that the work is there to be done, and the willing ones are so convinced of its necessity that they will choke in the collar rather than see it left undone. At the same time, however, they do not work with their eyes shut, but, on the contrary, see very plainly what a very large number of fat, sleek-coated creatures stand by, without so much as offering to draw even a single pound of the burden.

A Just Complaint.

It was once permitted to an ass to use articulate speech, and even to rebuke so great a personage as a prophet. Taking advantage of such venerable precedent, a horse, who has long suffered in silence, asks leave to lift up his voice. "Why, my prosperous and sleek-coated friend, do you stand all day knee-deep in rich clover whilst your brother quadruped is outside on the dusty road, drawing not only his own peculiar life-burden, but an additional load in the shape of charitable duty? Has it never occurred to you that the burden of charity is by no means a very light one in these populous and unthrifty times; and that very few are putting their shoulders to it—of which few you are not one? Are you aware that it is sometimes a sign of grace to feel ashamed of one's self, and don't you think that every well-conditioned quadruped ought to be ashamed of himself if he sees a brother striving hard to draw a heavy load up a steep hill and feels no unconquerable desire to jump over the hedge and help him?"

Meanness somewhere!

Seriously, the duty of furnishing money and other help for hospitals is taken up by a very limited number of people—how limited those only know who are familiar with the practical working of a hospital. The duty steadily increases with the steady increase of the population, but there seems to be no corresponding increase in the number of those who are willing to perform it. Why is this? There must be meanness somewhere. It cannot be the result of ignorance, because hospitals generally, and the London hospitals especially, with their magnitude of good works, occupy so large a space in public estimation, that no person who has eyes can fail to see and know all about them. Are people weary of hearing the constant cry of hospital managers for more money? How weary, then, do they think those managers themselves must be who, after giving all the money and time they can spare, without fee or reward, have so constantly to raise that cry?

The Duty of All.

It is the duty of every man who can spare a single shilling annually (and who cannot?) to spare it for these most noble and most useful institutions. If every man gave the smallest tithe, even of his superfluities, the hospitals would be rich. Let every well-conditioned horse who has hitherto contented himself with the enjoyment of his clover-field, leap over the hedge and place himself by the side of those willing animals who find their greatest contentment in taking their full share of all the duties and charities of life.

